# Evicted children and subsequent placement in out-of-home care: A cohort study

**DOI:** 10.1371/journal.pone.0195295

**Published:** 2018-04-18

**Authors:** Lisa Berg, Lars Brännström

**Affiliations:** 1 Centre for Health Equity Studies, Department of Public Health Sciences, Stockholm University, Stockholm, Sweden; 2 Swedish Institute for Social Research, Stockholm University, Stockholm, Sweden; 3 Department of Social Work, Stockholm University, Stockholm, Sweden; UNITED STATES

## Abstract

**Background:**

Evictions may have serious consequences for individuals’ health and wellbeing. Even though an eviction may be experienced as a significant crisis for the family, there is little previous knowledge on consequences for evicted children. This study represents the first attempt to examine to what extent children from evicted households were separated from their parents and placed in out-of-home care (foster family or residential care) using population-based data, net of observed confounding factors related to the socioeconomic and psychosocial circumstances of their parents.

**Methods:**

This study takes advantage of information from a Swedish national database, consisting of about 8 000 evicted individuals and a random sample of 770 000 individuals from the national population, linked to individual-level, longitudinal data from Swedish national registers. Our analytical sample consists of information for more than 250 000 children born in 1995–2008, including 2 224 children from evicted households. We used binary logistic regression based on the Karlson/Holm/Breen method to account for observed imbalances at baseline between evicted and non-evicted children.

**Results:**

Compared to non-evicted children, the crude odds ratio for placement in out-of-home care in evicted children was 12.10 (95% CI 8.54–17.14). Net of observed confounding factors related to the socioeconomic and psychosocial circumstances of the parents, evicted children had a twofold elevated risk of being placed in out-of-home care (odds ratio 2.26, 95% CI 1.55–3.27). Crude OR for evicted children in comparison with children under threat of eviction (eviction not formally executed) was 1.71 (95% CI 1.17–2.49) and adjusted OR 1.58 (95% CI 1.06–2.35).

**Conclusion:**

Children who experience eviction constitute a disadvantaged group and are at significant risk of being separated from their parents and placed in out-of-home care. These results demonstrate the importance of providing support for these children and their parents. Strategies to prevent households with children from being evicted seem to be an important and viable intervention path.

## Introduction

The links between housing, home, and wellbeing are well studied. Being a physical base for relationships, having a home is associated with general well-being and health outcomes as well as child development [1, 2]. The US financial crisis of 2007–2008, and the Eurozone crisis of 2010, have meant growing risks of severe housing problems among both usually established and vulnerable groups. Evictions, the focus of this study, are here understood as the involuntary removal of people from their homes, and are expected to have a wide range of negative personal and social consequences, particularly for children [3, 4]. Prior research has identified associations between severe housing problems, i.e. evictions and foreclosures, and decreased chances of decent and affordable housing, residential mobility, homelessness and unemployment [5–8], parenting stress and family disruption [3, 5], depression [9, 10], and suicide [11, 12]. It has also been shown that evictions typically hit individuals with few resources and poor health [4, 8, 13, 14]. While previous studies indicate that evictions have an adverse effect on health and wellbeing in adults [15], we know little about consequences for children in evicted households. However, previous research has demonstrated that evicted mothers are more likely to suffer from depression, experience material hardship and parenting stress [3]. An overlap between housing instability and household instability has also been demonstrated [16]. Instability in housing and family may have negative consequences for children and adolescents and influence development and health, wellbeing and behavior throughout the life course [2].

Targeting children who are abused or neglected by their parents or adolescents with serious conduct problems and delinquency, out-of-home care is a far-reaching intervention into family life [17]. In Sweden, the pronounced aim is to provide the child with better opportunities for development than in an adverse home environment. Even though the aim of an out-of-home placement is to provide these children with better opportunities, childhood experience of out-of-home care seems to be one of the strongest markers for compromised long-term health and psychosocial development that we know of [18–20].

Evictions increase vulnerability to homelessness [21], demonstrated to be associated with increased involvement by child protective services [22], and previous research has also shown that children in homeless families are at greater risk for out-of-home placements [23–25]. In one previous American study, inadequate housing, identified by interviews with caseworkers and caregivers, increased risk for out-of-home placement in families under investigation by child protective services [26]. The current study represents the first attempt to estimate the effect of eviction on subsequent risks of out-of-home placement into foster and residential care, using population-based data.

Using unique register data for more than 250 000 Swedish children born 1995–2008 (including around 2 200 evicted children), this study asks whether there is an association between evictions of households including children and subsequent separation from the parents and placement in out-of-home care (foster family or residential care). Being evicted may be perceived as a fundamental stressor which potentially poses serious limitations for parents to give their children a safe and stable upbringing [27]. Furthermore, since socioeconomic factors and individual psychosocial factors (such as psychiatric disorders and criminal offending) associated with risk of eviction, are strongly associated with increased risk for offspring placement in out-of-home care [13, 14, 28, 29], we also consider the importance of socioeconomic and psychosocial circumstances of the parents for this association.

## Material and methods

### Data material and study population

This study takes advantage of data from a new Swedish database covering all judicial processes (e.g. all stages in summary proceedings and the eviction process) registered by the Swedish Enforcement Authority [30]. The Enforcement authority is the only authority in Sweden sanctioned to execute home evictions. After a court order or a decision after a summary proceeding, the creditor can make an application at the Enforcement Authority for the judgment to be executed. The most common reason for eviction is rent arrears; other common reasons are repeated late payments and nuisance [30, 31].

In addition to information on individuals exposed to eviction, the database also includes a 10% random sample of individuals (n ≈ 770 000) not exposed to eviction, representative of the Swedish population (aged 16 years or older on December 31, 2012). Through use of the personal identification number assigned to all Swedish individuals at the time of birth or at the time of immigration, individual-level, longitudinal data from other national registers were linked to the information on the evicted and non-evicted individuals. The personal identification numbers are replaced by random reference numbers before data is made available to researchers, and all data are anonymous. The researchers did not have access to any information that could identify individuals in the dataset. The project was approved by the ethics committee in the Stockholm region before any records were linked (2014/24-31/5).

For the purpose of the current study, we used information on all applications for evictions and all executed evictions during 2009–2011 retrieved from the database described above. A pooled dataset was created, containing record-linked national register data (see Table 1 for a list of registers) for the evicted and the non-evicted (i.e. the 10% random sample) individuals [32]. Among the evicted individuals, families with children were identified through The Multi-generational register. The exposed group consisted of all children born in 1995–2008 who had a parent who was evicted in 2009–2011 (i.e. when the children were aged 1–16 years) (n = 2224). The Multi-Generational register was also used to identify families with children among the 10% representative sample. Children of individuals included in the 10% representative sample, who were born in 1995–2008, constituted the non-evicted group (n = 250 146). Children with previous experiences of placement in out-of-home care were not included in the study population (n = 4919).

**Table 1:** List of national registers that are combined in the study.

National Register	Variables	Years when data was available
Multi-Generation Register	Parental identification number	N/A
Register of the Total Population	Birth year Sex Country of birth	N/A
Cause of Death Register	Date of death	1991–2015
Register of Children and Young Persons Subjected to Child Welfare Measures	Out-of-home care	1960–2013
Hospital Discharge Register	Parental psychiatric disorders Parental alcohol disorders	1987–2014
Register of Court Convictions	Parental criminal convictions	1990–2013
Longitudinal Integration Database for Health Insurance and Labour Market Studies	Geographic residency Parental educational level Social assistance recipiency Single parent household/divorce	1990–2013

In Sweden, after a notice to quit a lease has been served and there has been a decision from a court procedure, the landlord can apply for the eviction to be executed. At this stage of the eviction process, about 30% of the evictions are executed [33]. Thus, a large number of individuals are served with an application for an execution of an eviction but the eviction is never executed. One explanation is that many tenants move without being formally evicted since an eviction reduces their chances of a new lease [30]. In additional analyses we analyzed information from children of individuals who received a notice of eviction in 2009–2011, but who were not formally evicted (n = 7847). We refer to this sample as children from families under threat of eviction.

### Placement in out-of-home care

The outcome measure was defined as a first (ever) placement in out-of-home care (foster family or residential care) in 2012–2013, according to the Swedish Register of Children and Young Persons Subjected to Child Welfare Measures. Information on placements was only available until 2013 and due to the short-term follow-up period, the current study does not address duration of placement.

### Covariates

We use a large number of observed background variables to control for confounding. To the extent unobserved confounding is related to observed confounding, our comprehensive controls indirectly control for unobservables [34]. Evictions are more common in resource-poor households, and previous studies have shown an overrepresentation of individuals with foreign origin among evicted individuals [35]. Low educational levels and poor economic resources are more common in parents of children placed in out-of-home care and previous research also suggests that the overrepresentation of children with a foreign background in out-of-home care can be attributed to socioeconomic differences [28]. The potential importance of socioeconomic factors was considered through adjustment for parental educational level and recipiency of social assistance. Social assistance recipiency was analyzed as a dichotomous indicator of whether the childhood household had received means-tested social assistance of any amount in 2008. A dichotomous indicator of whether the parents lived together in 2008 was also included in the analyses. Information on year of birth and sex of the child, country/region of birth of child and parent, and geographic residency/municipality was also included in the analyses. Covering the entire territory of the country, municipalities are the lower-level local government entity. Information on geographic residency was used in the analyses in two ways: included in the regression models as dummy variables for the 290 Swedish municipalities, (i.e. fixed effects), and as a categorical variable where the 290 municipalities were categorized as “city”, “town” and “rural” (see S1, S2 and S3 Tables describing ORs and CIs of all covariates).

A number of parental psychosocial factors may co-occur with both evictions and placement in out-of-home care [13, 14, 28, 29]. Parental psychiatric disorders were defined by at least one hospitalization with a diagnosis indicating psychiatric disorders and/or self-inflicted injuries during 1995–2008, according to the national Hospital Discharge Register. Information from this register was also used to identify parents with at least one hospitalization with an ICD diagnosis indicating alcohol or illicit drug use during 1995–2008. Parental criminality was defined as at least once having been convicted of a crime during 1995–2008, according to the Register of Court Convictions.

### Statistical analyses

In studies with the aim of assessing effects of exposures, several approaches could be employed. Often baseline characteristics of the individuals are imbalanced between exposed and non-exposed groups and adjustments need to be made. This can be accomplished either through appropriate regression modeling or by various forms of matching estimators. Regression modeling can be described as a particular sort of weighted matching estimator [36], and therefore the differences between regression and matching estimates are unlikely to be of major empirical importance. It has also been shown that propensity score methods gave similar results to traditional regression modeling in observational studies [37]. In the current study, we use regression analysis to account for observed imbalances between evicted and non-evicted children.

Logistic regression models based on the Karlson/Holm/Breen method were used to estimate odds ratios (OR) and corresponding 95% confidence intervals (CI) [38]. The KHB method ensures that the crude and adjusted coefficients presented are measured on the same scale [39]. Since the prevalence of first placement in out-of-home care was low in our population (less than 10%), estimated ORs may be interpreted as risk ratios [40].

We examined the risk of placement in out-of-home care for a) evicted children in comparison with non-evicted children, b) children from families under threat of eviction in comparison with non-evicted children, and c) evicted children in comparison with children under threat of eviction, in three separate regression models. The latter analysis may to some extent approximate the true effect of eviction beyond the selective processes that lead to eviction since it is reasonable to assume that this contrast should substantially mitigate the usual omitted variable bias.

Potential differences with regard to gender of the child was investigated by means of including an interaction term between gender and exposure. Evicted families often include younger children [41]. Since younger children are more likely to be placed in out-of-home care because of reasons related to parenting problems and maltreatment, and since this group of children tend to be a more socially selected [28], we were also interested in investigating whether age moderated the association. We analyzed age of the child at the time of the eviction categorized as 1–6 years, 7–12 years, and 13–17 years.

Our data have a three-level structure: children (level 1, n = 260 217) who are nested in families/households (level 2, n = 158 832) which in turn are nested in municipalities (level 3, n = 290). Viewing the nested data structure as nuisance that needs correction, we use cluster-robust standard errors to account for the within-family error correlation [42]. To account for the clustering within municipalities, we use fixed-effects (dummy variables) which absorb all of the variation that occurs between these administrative units. When including all but one municipality-dummy in the model then there cannot be any between municipality variation explained by municipality-level variables such as variation in child welfare practices and preventive measures regarding evictions.

Stata version 14.2 was used for all analyses, the KHB-command was utilized for the logistic regression. The vce(cluster) option was used to account for family clustering [39].

## Results

Descriptive statistics are given in Table 2. The majority of the evicted children, 80%, were twelve years or younger at the time of the eviction. There were considerable differences with regard to parental educational level between the children who were formally evicted and the non-evicted children; 13.6% of the mothers and 10.2% of the fathers of the evicted children had a university education, compared with 45.2% of the mothers and 36.9% of the fathers in the non-evicted group. Having received means-tested social assistance was almost seven times more common among the parents of the evicted children. Two out of three of the evicted children had parents who did not live together, compared with one in six children in the non-evicted group. Having a parent who had been convicted of a crime was three to four times more common among the evicted children. Only minor differences were seen between the two groups with regard to hospitalizations among the parents. When compared with non-evicted children from the general population, children from families under threat of eviction were in many respects similar to the evicted children (Table 2).

**Table 2:** Characteristics of the study population.

	Non-evicted children	Evicted children	Children under threat of eviction
**Number of individuals**	250 146	2 224	7 847
**Sex**			
Boys	51.6	54.1	51.6
Girls	48.4	45.9	48.4
**Age at eviction**			
1–6 years	-	36.8	36.7
7–12 years	-	43.7	41.7
13–18 years	-	19.5	21.6
**Geographic residency**			
City	35.1	30.4	22.7
Town	37.1	38.2	42.5
Rural	27.8	31.4	34.8
**Country of birth**			
Sweden	96.1	93.5	93.4
European	1.6	3.2	3.3
Non-European	2.3	3.4	3.3
**Mother’s country of birth**			
Sweden	79.6	65.6	66.4
European	7.4	11.8	12.6
Non-European	13.0	22.6	21.0
**Father’s country of birth**			
Sweden	79.5	61.1	63.7
European	7.8	13.0	14.0
Non-European	12.7	25.9	22.3
**Mother educational level**			
Missing	1.7	6.1	5.7
Compulsory school	9.2	34.2	32.0
Secondary school	43.9	46.1	49.9
University	45.2	13.6	12.4
**Father educational level**			
Missing	1.5	4.2	4.2
Compulsory school	11.8	33.2	27.4
Secondary school	49.7	52.4	56.7
University	36.9	10.2	11.7
**Social assistance recipiency**			
Mother	5.3	35.2	33.4
Father	4.6	31.0	25.1
**Indicators of parental psychosocial problems**			
Mother criminal offending	4.7	20.9	17.2
Father criminal offending	17.9	57.2	45.6
Mother psychiatric disorder	4.1	4.1	4.2
Father Psychiatric disorder	4.0	4.1	3.9
Mother substance abuse	3.1	2.9	3.0
Father substance abuse	3.2	3.3	2.7
**Family composition**			
Parents separated/divorced	16.2	67.6	59.4

Among the evicted children, 3.6% were placed in out-of-home care during the two-year follow-up period, compared with 0.3% among non-evicted children during the same time period. The evicted children were younger at the time of the first placement (Table 3). Among children from families under threat of eviction, 2.0% were placed in out-of-home care during the two year period.

**Table 3:** Out-of-home care in 2012–2013.

	Non-evicted children	Evicted children	Children under threat of eviction
**First placement during 2012–13**	867 (0.3%)	80 (3.6%)	160 (2.0%)
**Age at first placement**			
4–6 years old	88 (10.2%)	16 (20.0%)	13 (8.1%)
7–12 years old	223 (25.7%)	33 (41.3%)	59 (36.9%)
13–18 years old	556 (64.1%)	31 (38.8%)	88 (55.0%)

Crude OR for a first placement in out-of-home for the evicted children, compared with children from the comparison group was 12.10 (95% CI 8.54–17.14). Although OR was substantially reduced in the fully adjusted model, the risk of a being placed in out-of-home care was more than twice as high for evicted children (Table 4). Elevated ORs for out-of-home placement were also seen in analyses of children from households under threat of eviction and for evicted children in comparison with children under threat of eviction (Table 4).

**Table 4:** ORs of a first placement in out-of-home care during 2012–2013.

		OR	95% CI
**Evicted children (ref non-evicted children)**	Crude	12.10	8.54–17.14
	Adjusted^1^	2.26	1.55–3.30
**Children under threat of eviction (ref non-evicted children)**	Crude	6.89	5.55–8.55
	Adjusted^1^	1.65	1.30–2.10
**Evicted children (ref children under threat of eviction)**	Crude	1.71	1.17–2.49
	Adjusted^1^	1.58	1.06–2.35

^1^ Adjusted for year of birth, sex, country of birth of parent and child, geographic residency, municipality dummies (fixed effects), parental educational level, recipiency of social assistance, family composition, parental hospitalizations for psychiatric and substance abuse related disorders, and parental criminality. See Supplemental tables (S1, S2 and S3 Tables) for estimates for the control variables.

Interaction analyses indicated no effect modification by sex (p >0.05). We also analyzed the importance of age of the child at the time of eviction and the adjusted ORs in the analyses comparing evicted children with non-evicted children were 3.44, 95% CI 2.03–5.86 (0–6 years), 2.09, 95% CI 1.30 3.36 (7–12 years) and 1.56, 95% CI 0.88–2.78 (13–16 years).

## Discussion

To our knowledge, this is the first population-based study to longitudinally investigate risk of placement in out-of-home care for children from recently evicted households. Evicted children had a remarkably increased risk of a first placement, compared with children from the general population. A large part of the elevated OR was accounted for by observed confounding factors related to parental socioeconomic and psychosocial circumstances. Yet, after adjustments, the risk of placement in out-of-home care was twice as high for the evicted children.

Our results indicate that children from evicted households constitute a vulnerable group. Evictions were, in particular, associated with low educational levels, high proportions of social assistance recipiency and high levels of criminal offending in parents. In agreement with previous findings of strong links between socioeconomic disadvantage [28, 29] and parental incarceration [43, 44] and risk of placement in out-of-home care, these parental factors were found to explain part of the associations in our data. Previously it has been demonstrated housing instability is associated with household instability, i.e. changes in family composition and family dissolution [16], and single parenthood has previously been shown to be a strong risk factor for care placement [28]. In the present study, not living with both parents was far more common among the evicted children and contributed to the differences seen between the evicted and non-evicted children. In addition to socioeconomic and psychosocial problems existing already before the eviction, it is reasonable to assume that the eviction process and its consequences put additional strain on the family and may cause increased parental stress [45, 46]. Increased parental stress may lead to less capacity for good parenting, less ability to provide children with social and emotional support and a safe and stable upbringing. Previous studies have demonstrated links between housing insecurity and increased risk of child maltreatment and abuse, both directly and through parental stress [27, 47]. Inadequate care and child maltreatment are main reasons for placement of younger children in care [28]. In our data, more than one third of the evicted children were younger than 6 years at the time of eviction and the children in this age group were more likely to be placed in care, compared to children who were older at the time of eviction. Among the evicted children, one in every five new placements was preschool children and among the non-evicted children the corresponding number was one in every ten children.

In Sweden, a considerable number of households receive a notice of eviction although the eviction is never formally executed; about 30% of the for execution applied evictions are executed [33]. Our results demonstrate an increased risk of out-of-home placement also in children from households under threat of eviction. As for evicted families, these associations may in part be explained by pre-existing parental and familial factors as well as by the increased strain put on the family and the parents from the threat of an imminent eviction. A previous study based on the same data material indicated that the mere prospect of losing one’s home, e.g. the threat of eviction, may be experienced as a significant crisis and increase suicide risk [11]. In addition, when families are threatened with eviction the family may choose to hastily leave their accommodation and many of these informally forced moves may be comparable to an actual eviction [31]. The number of such informally forced moves triggered by the eviction threat is unknown, but previous interview studies among evictees in Sweden suggest that at least half of the interviewed individuals had left the dwellings before the actual eviction date [48]. In additional analyses, evicted children were compared with children under threat of eviction. In this attempt to more closely approximate the effect of the eviction per se, an increased risk was seen for the evicted children also in this comparison with a group of children who were more similar each other with regard to important parental and household characteristics.

As a worst case scenario for the family, evictions may lead to homelessness [5, 7]. In an American context, it has been demonstrated that substantial proportions of homeless individuals are separated from their children and that the children live with family and friends or in foster care [23, 25, 49]. One previous cross-sectional study of 195 foster children, demonstrated that almost half of the birth parents had experienced homelessness [50]. However, longitudinal data have been lacking and our study contributes with new insight into the association between evictions and its consequences for children.

### Strengths and limitations

The strengths of this study include the large cohort sample (including a sizeable number of children who had experienced an eviction), the use of national register data with low attrition, comprehensive controls of robust socioeconomic and psychosocial confounders related to the parents, and temporal ordering of the variables of primary interest.

Although our comprehensive set of controls of observed confounders may have bought some protection against confounding by unobservables [34], data that could be vital for our understanding of the links between family evictions and subsequent placement in out-of-home care are often outside the scope of register-based studies. For example, the Swedish child welfare register does not contain data on reasons for placement in out-of-home care (e.g. abuse or neglect). We also do not have information on homelessness, e.g. usage of shelters, or repeated residential changes. We further do not have data on the hypothesized link between exposure to deprived residential neighborhoods and child maltreatment/parenting styles [51, 52], although a number of studies based on individual-level data suggest that this link is probably non-causal [53, 54]. Another limitation is that children with separated or divorced parents are recorded as living with just one of the parents, and we cannot know to what extent children from non-intact families were living with only one parent or alternately with both parents. However, the proportion of children in joint physical custody, i.e. where children alter their residency between the parents’ homes, is high in Sweden; 35–40% of all children from non-intact families spend more or less equal time with both parents [55], and previous studies have also shown that a majority of Swedish children have regular contact with both parents even if the parents do not live together [56]. Furthermore, a previous Swedish study showed that in 75% of the families under threat of eviction the child lived permanently in the dwelling from which the tenants are evicted [57]. According to Swedish law, the landlord is obliged to inform the local social welfare board when the tenant has been served with a notice to quit and the lease has been terminated, and the social welfare board must also be informed by the Enforcement Authority after the formal decision for the eviction to be executed. Thus, another potential limitation is the possibility that children from evicted families who are abused or neglected to a greater extent are detected by social services, compared to children in the comparison group. We have, however, excluded children with previous placements in out-of-home care, and the strength of the associations are therefore more likely to be underestimated, rather than the opposite.

### Implications

The findings of the present study indicate that children who experience an eviction constitute a disadvantaged group in the national population and that these children are at significant risk of being placed in out-of-home care. Out-of-home placements were consistently more common in evicted children compared to children from non-evicted families with similar characteristics, indicating that eviction adds to the risk in already vulnerable families. Recently published studies emphasize the vulnerability of children placed in foster care, both as a result of early life circumstances but also since foster care placement in itself may be a risk factor for health problems in childhood [58–60]. Furthermore, even though the aim of an out-of-home placement is to provide children from adverse birth homes with better opportunities, these individuals constitute a group with a particular high risk of negative long term outcomes [19, 20]. Former foster youth also experience increased risks of homelessness, low educational attainment and unemployment [61]. Our findings emphasize the need of support for evicted children and their parents and the importance of addressing underlying economic and psychosocial problems in families at risk of eviction. A recent report from the European Commission [21] highlights a number of prevention measures, both general macro-level housing policies but also secondary prevention measures directed at individuals with a potentially high risk of eviction and homelessness, including counselling, support through social services and public assistance with housing costs and rent arrears. Avoiding households with children from being evicted, e.g. through such preventive policies, seems to be an important and viable intervention path.

### Conclusion

Our results demonstrated that evicted children had a twofold elevated risk of being placed in out-of-home care, compared with children from the general population net of socioeconomic and psychosocial circumstances of the parents. These findings emphasize the need of support for these children and their parents, the importance of addressing underlying economic and psychosocial problems in families at risk of eviction, and of developing strategies focusing on avoiding households with children from being evicted.

## Supporting information

S1 Table Odds ratios (OR) and 95% confidence intervals (CI) for control variables related to the adjusted logistic regression analysis for evicted children vs. non-evicted children reported in Table 4 (intercept and 289 municipality dummies suppressed). (DOCX)

S2 Table Odds ratios (OR) and 95% confidence intervals (CI) for control variables related to the adjusted analysis for children under threat of eviction vs. non-evicted children reported in Table 4 (intercept and 289 municipality dummies suppressed). (DOCX)

S3 Table Odds ratios (OR) and 95% confidence intervals (CI) for control variables related to the adjusted analysis for evicted children vs. children under threat of eviction reported in Table 4 (intercept and 289 municipality dummies suppressed). (DOCX)
