# Supplementary material for: Evicted children and subsequent placement in out-of-home care: A cohort study
Source: PLoS One. 2018 Apr 18;13(4):e0195295. doi: 10.1371/journal.pone.0195295 (PMC5905888; doi:10.1371/journal.pone.0195295)
Supplement: S1 Table — (DOCX) [file pone.0195295.s001.docx]

S1 Table. Odds ratios (OR) and 95% confidence intervals (CI) for control variables related to the adjusted logistic regression analysis for evicted children vs. non-evicted children reported in Table 4 (intercept and 289 municipality dummies suppressed).

|  | OR | 95% CI |
| --- | --- | --- |
| Girl | 1.12 | 0.98-1.29 |
|  |  |  |
| Birth year (cont.) | 0.90 | 0.88-0.92 |
|  |  |  |
| Born in Sweden | 0.97 | 0.66-1.42 |
|  |  |  |
| Geographic residency: City (ref.) |  |  |
| Geographic residency: Town | 2.76 | 0.71-10.68 |
| Geographic residency: Rural | 2.84 | 0.60-13.46 |
|  |  |  |
| Mother compulsory school (ref.) |  |  |
| Mother secondary school | 0.70 | 0.57-0.87 |
| Mother university | 0.37 | 0.27-0.49 |
|  |  |  |
| Father compulsory school (ref.) |  |  |
| Father secondary school | 0.87 | 0.71-1.07 |
| Father university | 0.72 | 0.54-0.95 |
|  |  |  |
| Mother’s country of birth: Sweden (ref.) |  |  |
| Mother’s country of birth: European | 0.91 | 0.62-1.34 |
| Mother’s country of birth: Non-European | 1.09 | 0.75-1.61 |
|  |  |  |
| Father’s country of birth: Sweden (ref.) |  |  |
| Father’s country of birth: European | 1.45 | 1.02-2.06 |
| Father’s country of birth: Non-European | 1.30 | 0.90-1.88 |
|  |  |  |
| Mother social assistance recipiency | 2.00 | 1.54-2.59 |
| Father social assistance recipiency | 1.58 | 1.22-2.04 |
|  |  |  |
| Mother criminal offending | 1.87 | 1.49-2.36 |
| Father criminal offending | 2.10 | 1.74-2.53 |
|  |  |  |
| Mother psychiatric disorder | 0.96 | 0.62-1.46 |
| Father psychiatric disorder | 0.89 | 0.58-1.37 |
|  |  |  |
| Mother substance abuse | 1.15 | 0.69-1.91 |
| Father substance abuse | 0.88 | 0.55-1.42 |
|  |  |  |
| Parents separated/divorced | 2.48 | 2.08-2.97 |
