# Supplementary material for: Evicted children and subsequent placement in out-of-home care: A cohort study
Source: PLoS One. 2018 Apr 18;13(4):e0195295. doi: 10.1371/journal.pone.0195295 (PMC5905888; doi:10.1371/journal.pone.0195295)
Supplement: S2 Table — (DOCX) [file pone.0195295.s002.docx]

S2 Table. Odds ratios (OR) and 95% confidence intervals (CI) for control variables related to the adjusted analysis for children under threat of eviction vs. non-evicted children reported in Table 4 (intercept and 289 municipality dummies suppressed).

|  | OR | 95% CI |
| --- | --- | --- |
| Girl | 1.08 | 0.95-1.23 |
|  |  |  |
| Birth year (cont.) | 0.90 | 0.88-0.92 |
|  |  |  |
| Born in Sweden | 0.89 | 0.62-1.27 |
|  |  |  |
| Geographic residency: City (ref.) |  |  |
| Geographic residency: Town | 2.74 | 0.97-7.74 |
| Geographic residency: Rural | 1.27 | 0.32-4.98 |
|  |  |  |
| Mother compulsory school (ref.) |  |  |
| Mother secondary school | 0.76 | 0.62-0.93 |
| Mother university | 0.38 | 0.28-0.50 |
|  |  |  |
| Father compulsory school (ref.) |  |  |
| Father secondary school | 0.88 | 0.73-1.07 |
| Father university | 0.74 | 0.57-0.97 |
|  |  |  |
| Mother’s country of birth: Sweden (ref.) |  |  |
| Mother’s country of birth: European | 0.90 | 0.62-1.30 |
| Mother’s country of birth: Non-European | 1.18 | 0.82-1.68 |
|  |  |  |
| Father’s country of birth: Sweden (ref.) |  |  |
| Father’s country of birth: European | 1.43 | 1.02-2.00 |
| Father’s country of birth: Non-European | 1.35 | 0.95-1.91 |
|  |  |  |
| Mother social assistance recipiency | 1.99 | 1.57-2.51 |
| Father social assistance recipiency | 1.49 | 1.17-1.89 |
|  |  |  |
| Mother criminal offending | 1.84 | 1.49-2.28 |
| Father criminal offending | 1.96 | 1.65-2.33 |
|  |  |  |
| Mother psychiatric disorder | 0.98 | 0.66-1.44 |
| Father psychiatric disorder | 0.78 | 0.51-1.20 |
|  |  |  |
| Mother substance abuse | 1.14 | 0.71-1.83 |
| Father substance abuse | 0.91 | 0.57-1.44 |
|  |  |  |
| Parents separated/divorced | 2.60 | 2.20-3.07 |
