# Supplementary material for: Evicted children and subsequent placement in out-of-home care: A cohort study
Source: PLoS One. 2018 Apr 18;13(4):e0195295. doi: 10.1371/journal.pone.0195295 (PMC5905888; doi:10.1371/journal.pone.0195295)
Supplement: S3 Table — (DOCX) [file pone.0195295.s003.docx]

S3 Table. Odds ratios (OR) and 95% confidence intervals (CI) for control variables related to the adjusted analysis for evicted children vs. children under threat of eviction reported in Table 4 (intercept and 289 municipality dummies suppressed).

|  | OR | 95% CI |
| --- | --- | --- |
| Girl | 0.95 | 0.72-1.26 |
|  |  |  |
| Birth year (cont.) | 0.93 | 0.90-0.97 |
|  |  |  |
| Born in Sweden | 0.51 | 0.24-1.10 |
|  |  |  |
| Geographic residency: City (ref.) |  |  |
| Geographic residency: Town | 0.87 | 0.57-1.35 |
| Geographic residency: Rural | 0.88 | 0.56-1.35 |
|  |  |  |
| Mother compulsory school (ref.) |  |  |
| Mother secondary school | 0.66 | 0.47-0.95 |
| Mother university | 0.48 | 0.26-0.88 |
|  |  |  |
| Father compulsory school (ref.) |  |  |
| Father secondary school | 1.18 | 0.80-1.74 |
| Father university | 0.80 | 0.42-1.53 |
|  |  |  |
| Mother’s country of birth: Sweden (ref.) |  |  |
| Mother’s country of birth: European | 0.91 | 0.46-1.80 |
| Mother’s country of birth: Non-European | 0.49 | 0.25-0.95 |
|  |  |  |
| Father’s country of birth: Sweden (ref.) |  |  |
| Father’s country of birth: European | 0.93 | 0.49-1.76 |
| Father’s country of birth: Non-European | 1.27 | 0.72-2.26 |
|  |  |  |
| Mother social assistance recipiency | 2.12 | 1.48-3.02 |
| Father social assistance recipiency | 1.09 | 0.76-1.57 |
|  |  |  |
| Mother criminal offending | 1.32 | 0.91-1.92 |
| Father criminal offending | 1.40 | 1.00-1.96 |
|  |  |  |
| Mother psychiatric disorder | 0.44 | 0.17-1.14 |
| Father psychiatric disorder | 0.35 | 0.10-1.24 |
|  |  |  |
| Mother substance abuse | 1.93 | 0.86-4.35 |
| Father substance abuse | 0.61 | 0.18-2.10 |
|  |  |  |
| Parents separated/divorced | 0.93 | 0.63-1.38 |
